# Molecular and Phylogenetic Analyses Suggest an Additional Hepatitis B Virus Genotype “I”

**DOI:** 10.1371/journal.pone.0009297

**Published:** 2010-02-19

**Authors:** Hai Yu, Quan Yuan, Sheng-Xiang Ge, Hurng-Yi Wang, Ya-Li Zhang, Qing-Rui Chen, Jun Zhang, Pei-Jer Chen, Ning-Shao Xia

**Affiliations:** 1 National Institute of Diagnostics and Vaccine Development in Infectious Diseases, Xiamen University, Xiamen, China; 2 Graduate Insititute of Clinical Medicine, National Taiwan University and Hospital, Taipei, Taiwan; Yonsei University, Republic of Korea

## Abstract

A novel hepatitis B virus (HBV) strain (W29) was isolated from serum samples in the northwest of China. Phylogenetic and distance analyses indicate that this strain is grouped with a series of distinct strains discovered in Vietnam and Laos that have been proposed to be a new genotype I. TreeOrderScan and GroupScan methods were used to study the intergenotype recombination of this special group. Recombination plots and tree maps of W29 and these putative genotype I strains exhibit distinct characteristics that are unexpected in typical genotype C strains of HBV. The amino acids of P gene, S gene, X gene, and C gene of all genotypes (including subtypes) were compared, and eight unique sites were found in genotype I. In vitro and in vivo experiments were also conducted to determine phenotypic characteristics between W29 and other representative strains of different genotypes obtained from China. Secretion of HBsAg in Huh7 cells is uniformly abundant among genotypes A, B, C, and I (W29), but not genotype D. HBeAg secretion is low in genotype I (W29), whose level is close to genotype A and much lower than genotypes B, C, and D. Results from the acute hydrodynamic injection mouse model also exhibit a similar pattern. From an overview of the results, the viral markers of W29 (I1) in Huh7 cells and mice had a more similar level to genotype A than genotype C, although the latter was closer to W29 in distance analysis. All evidence suggests that W29, together with other related strains found in Vietnam and Laos, should be classified into a new genotype.

## Introduction

The hepatitis B virus (HBV) is globally distributed, infecting approximately one-third of the world's human population. Genotypes of HBV were first suggested by Okamoto who noted that 4 HBV genomes of different genotype differed by more than 8% [1]. Then threshold had been used for separation of the different genotypes. With more and more HBV complete sequence discovered, a threshold of 7.5% was suggested after a phylogenetic analysis and pairwise comparison of 670 complete genomes [2]. Norder also suggested to take the first branch (S gene) in an alignment to assign genotypes [3]. Presently, eight genotypes of HBV, designated with A to H, have been recognized [4], [5], [6]. Some of the genotypes could be mapped to a special geographic distribution but others could not. Consider not varying phrasing so often: Genotype A is prevalent in sub-Saharan Africa, North America and Europe; B/C, in Asia; E, in Africa; and F/H, in Central and South America. Genotypes D/G, on the other hand, seem to be scattered worldwide. In addition to these acknowledged genotypes, there're putative genotypes that could not classify into those group above. Such variants include genotype I [5], [7] and J [8]. Prior to the definition of genotypes, HBV stains can be divided into 9 serotypes, designated as *ayw1*, *ayw2*, *ayw3*, *ayw4*, *ayr*, *adw2*, *adw4q−*, *adrq+*, and *adrq−*, according to the different serological analysis results of surface antigen [9], [10]. Although there are some complicated relationships between genotypes and serotypes in HBV, increasing evidence shows that genotypes play a greater role in determining the clinical severity and development of chronic infection [11]. Furthermore, the occurrence of HBeAg negative chronic hepatitis B is also genotype-specific related [11].

The first HBV viral particles were produced *in vitro* through transfecting cultured cells with a linear tandem dimer of HBV [12]. Virions thus produced are morphologically and virologically indistinguishable from the authentic virion [13], and can infect chimpanzees [14]. The method was supplemented by the creation of a a modified, extended genome (∼1.3 fold) for transfection which could be transcribed into overlength pregenomic and precore mRNAs, providing an *in vitro* model for studying HBV infections. *In vivo* studies had been hampered by a lack of suitably small and well-characterized animals. Eventually, a mouse model of acute HBV infection based on hydrodynamic injection and supporting transitory HBV replication was successfully developed. This model allowed the examination of HBV dynamics in a tightly controlled *in vivo* system [15], [16].

In this study, we isolate a distinct HBV strain (W29) in the northwest of China. Phylogenetic analysis indicates that this strain is grouped with a series of distinct strains discovered in Vietnam and Laos [7], [17]. The latter have been proposed to be classified as a new genotype. A new analysis method was adopted to compare the recombination of W29 with the putative new genotype I strains, as well as other known genotypes. The methods TreeOrderScan and GroupScan were first implemented by Simmond [18] who designed them to identify independent intergenotype recombinants of HBV using a series of novel phylogenetic and bioinformatics analysis methods. We compared the recombination plot of W29 with that of the putative new genotype I strains, and other known genotypes as well, and found that W29 and the genotype I strains exhibit some distinct characteristics which are unexpected in the regular genotype C strains. The amino acids sequences of P, S, X and C gene of all genotypes (including subtypes) were compared to illustrate some unique sites in genotype I. *In vitro and in vivo* experiments were also conducted to compare phenotypic characteristics between W29 and other genotypes obtained from China. All evidence suggests that the W29, together with other related strains found in Vietnam and Laos, could be classified into a new genotype.

## Methods

### Ethics Statement

The study was approved by the Human Subject Committees at You An Hospital in Beijing, Xi Jing Hospital in Xi'an and Xiamen Centers for Disease Control. Written informed consent was obtained from all subjects.

### Serum Sample

The W29 case was a 23-year-old female asymptomatic HBV carrier who had never left the mainland and was living in Xi'an in the northwest China. The W29 serum sample was obtained when she received her annual checkup at the hospital in 2006. Serum samples of genotypes Ae and Ba (N10 and C4371, respectively) were obtained from two HBV carriers living in Fujian province in southeast China. The sample of genotype C1 (Y1021) was obtained from a carrier in Beijing in northern China. The sample of genotype D1 (Y10) was obtained from a carrier in Xi'an in northwest China. All five sera were HBeAg-positive with detectable levels of HBV DNA, and without the precore G1896A or core promoter A1762T/G1764A mutations that may affect HBeAg expression and viral replication.

### HBV Full-Length Genome Sequencing

HBV DNA was extracted from 100 µL of serum using QIAamp DNA blood kits (QIAGEN, Hilden, Germany). Complete HBV genomes were amplified by a “hot-start” polymerase chain reaction using the previously reported method [19]. The amplified fragments (−1825∼+1821) were sequenced on an ABI Prism 3130X automatic genetic analyzer, then inserted into pGEM-T Easy Vector (Takara, Dalian, China) and cloned in DH5α competent cells. The clones which shared consensus sequences with the PCR-product were used to construct 1.35-fold genome plasmids.

### Reference Sequences

All available complete genome sequences of HBV were downloaded from GenBank at April 2009 with identical entries excluded. Multiple alignment was done with ClustalX2 under default settings. Subsequent sequence manipulation was carried out in the Simmonic sequence editor package [18] (available from http://www.polio.vir.gla.ac.uk). To make the sequence set most representative and informative in terms of phylogeny, the most related sequences were first removed from the dataset with all pairwise distance scan. Genotype information was extracted from the GenBank definition, noted automatically and each known sequence was tagged. In this way, recombinant sequences were removed. Nearly two-thirds of all sequences were assigned with a genotype tag, and these were designated as set A. The remainder were temporarily designated as set B. GroupScan Analysis (described below) using the known set A for each sequence in Set A and B was carried out by batch command. With this self-optimization method, potential recombinant forms in set A were picked out according to the output plot of GroupScan and non-recombinant forms in set B were recruited into the dataset. For new sequences added, pairwise distance scan was carried out again to exclude the most related sequences and the final dataset contained 322 sequences with genotype tag as A-H and Chimpanzee. Subtype information was extracted from related articles and by further phylogenic analysis. All aligned reference sequences with subtype tags were then truncated and translated into P, X, C and S amino acid sequences. Consensus sequences of each sub-genotype group of HBV were concluded first. This was done by examining every nucleotide position of all the aligned sequences in one group for a most abundant one. Then these consensus sequences were put together and mark with different colors using MS Excel software to screen for the unique site in each subtype.

### Phylogenetic and Recombination Analysis

Our newly discovered strain W29, together with three sequences described as ‘aberrant HBV Vietnamese strains’ (AB231908, AF241408, and AF241409) [20] and the other two sequences from Laos (FJ023661, FJ023663) [17] for comparison were selected for phylogenetic and recombination analysis. Distance Calculation within predefined groups was done with the MEGA4 package [21]. A Tree containing these strains and the reference sequences as described above was constructed by neighbor-joining with Maximum Composite Likelihood corrected distances in the MEGA4 package [21], using 500 bootstrap replicates. Nucleotide sequences of the P gene were also submitted to MEGA4 and a tree was created using the same method. GroupScan Analysis in the Simmonic software package was used to plot the recombinant constitution of W29 along with other five related sequences (window = 200, step = 50). TreeOrderScan was also carried out in Simmonic with genotype I sequences included as an individual group and the output data were grouped and plotted as previous publication [18].

### HBV 1.35-Fold Genome Plasmid Constructs

A 1.35-fold-overlength genome of HBV was constructed via overlap extension PCRs and inserted into a CMV-promoter-deleted pcDNA3.1 vector (Invitrogen, Shanghai, China, digested by Spe I endonuclease). The genome had a 5′ terminal redundancy starting at −961 nt and a 3′ terminal end at +2000 nt, including the entire polyadenylation site. The body of the genome included Enh I and II, the X- and pregenomic/core promoter regions, and the origin of replication (DR I and II). All five clones of genotypes A, B, C and D were submitted to GroupScan to evaluate the genotype representative before they were annealed into plasmid constructs. Each HBV 1.35-fold genome clone was confirmed by DNA sequencing.

### Cell Culture and In Vitro Transfection

The plasmids used for *in vitro* transfection were purified with PlasmidSelect Xtra Starter Kit (GE health, Sweden) and the concentrations determined by the UV-spectrophotometric method. After 24 hours of culture, Huh7 cells were transfected with 5 µg of DNA construct using Lipofectamine 2000 (GBICO, USA) and harvested 4 days later. Transfection efficiency was measured by cotransfection with 0.1 µg of a reporter plasmid expressing secreted alkaline phosphatase (SEAP) and estimating SEAP enzymatic activity in the culture supernatant. Triple experiments were conducted for each clone.

### Hydrodynamic Injection of Plasmid to Mice

To compare the *in vivo* HBV marker levels in different genotypes, an HBV hydrodynamic injection assay was conducted using 6–8 weeks old BALB/C mice. In brief, 40 µg of purified plasmid was diluted to 2 mL with physiological saline and injected into tail vein within 5–10 s. Mice sera were assayed for HBsAg and HBeAg at 0∼9 days after injection. For each group, five mice were used. All animals received humane care and the study protocol is complied with the institution's guidelines.

### Determination of HBV Markers in the Culture Supernatant and Mouse Serum

HBsAg, HBeAg and HBcAg levels were determined by chemiluminescence using commercial assay kits (Wantai, Beijing, China). The relative level of each antigen was expressed as an S/CO ratio, on a linear range from 1 to 1000 for all three assays. The lower detection limit was 10pg/ml for the HBsAg and HBeAg assays, and 50 pg/ml for the HBcAg. For extracellular HBV DNA assay, the collected supernatant was treated with 200 µg/mL DNase I and 100 µg/mL RNase A at 37°C for 2 hours, with reaction terminated by EDTA at a final concentration of 10 mM. The mixture was then incubated at 65°C for 10 min and the HBV DNA was extracted using QIAamp DNA blood kits (QIAGEN, Hilden, Germany). HBV DNA quantification assays were performed using a commercial real-time PCR kit (Kehua, Shanghai, China).

### Nucleotide Sequence Accession Numbers

The nucleotide sequence data reported in this paper will appear in the DDBJ/EMBL/GenBank databases under the accession AY707087 N10 Ae, GU357842 C4371 Ba, GU357845 Y1021 C1, GU357846 Y10 D1, and GU357844 W29 I1.

## Results

### W29 Is More Related to the Putative New Genotype I Group

**Figure 1:**
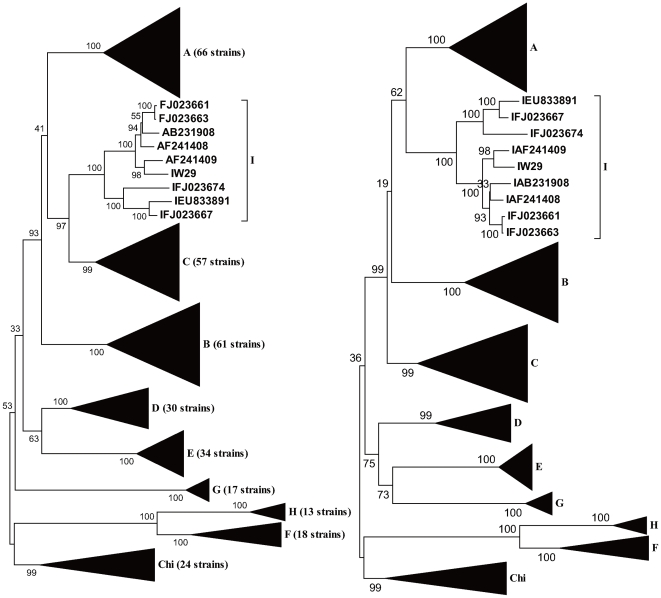
Phylogenetic analysis of complete genomes (left) and P gene products (right). The sequences include all the reference sequences, representative Vietnam and Laos' sequence and W29 strains. The putative genotype I strains are separated as an independent clade. Sequences of Genotype A-H have been collapsed into black triangles.

**Figure 2:**
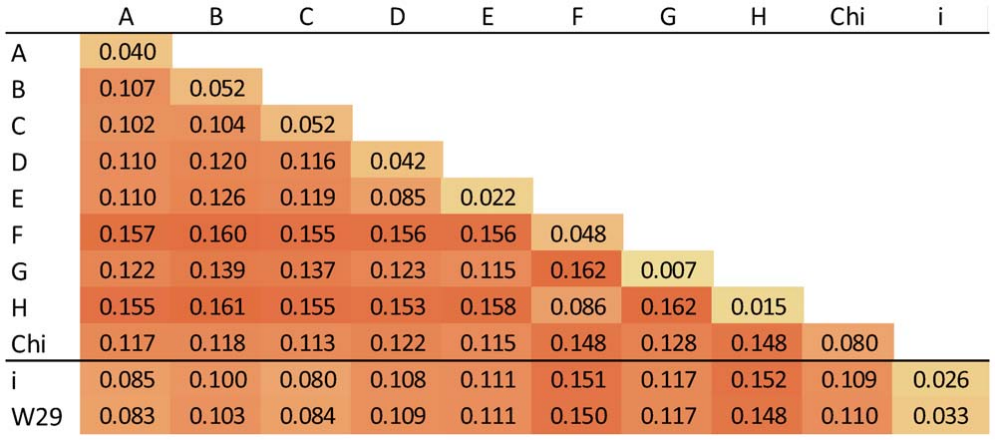
Mean distances between full-length sequence of genotype A-H, genotype chimpanzee (Chi) and genotype I, generated by MEGA4 package. Values on the diagonal are the mean distances within each genotype. The calculation is based on the Maximum Composite Likelihood distance correction method.

Leaving out W29, the other four strains can be clearly identified as Genotype A, B, C and D (Data not shown). Phylogenetic trees were generated using complete genomes and amino acid sequences of P gene products, and both trees show that W29 falls within the same sequence clade as the Vietnam and Laos strains, with this clade being separated from genotype C groups (Fig. 1). The suggestion that this clade is a new genotype of HBV is still under dispute [17], [20], [22]. In the tree of P gene, the clade seems more related to genotype A than genotype C. Treating the new genotype as genotype I, the grouping of W29 with Vietnam and Laos' sequence was confirmed by distance calculation (Fig. 2). Distance values between the I *group* and other genotypes are no less than 0.08, and the distance between W29 and I group is only 0.033 which is close to intra-genotype distance values. Furthermore, the distance between I *group* and genotype A/C (∼0.08) is smaller than other groups, which is consistent with the phylogenetic tree result.

### Recombination Analysis of W29 and Other Related Sequences

**Figure 3:**
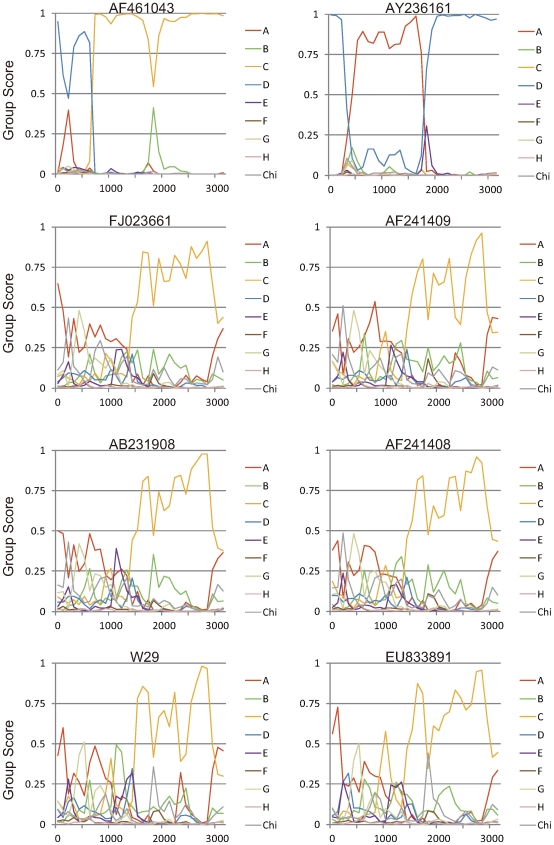
Group Scan Analysis. Eight HBV strains were submitted to Group Scan program and calculated against the reference groups of nonrecombinant HBV sequences from human genotypes A to H and nonhuman ape-derived variants (*n* = 322) [18].

**Figure 4:**
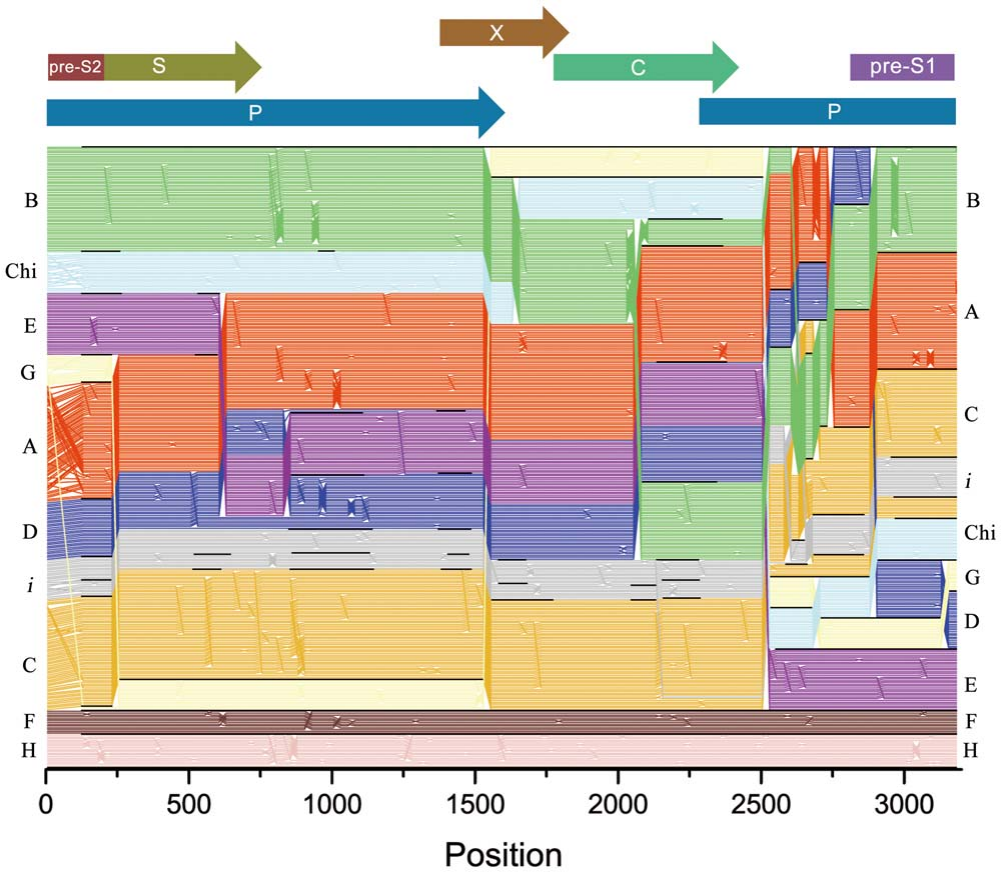
TreeOrder Scan of nonrecombinant HBV sequences. It shows positions of individual sequences (y axis) in phylogenetic trees generated from sequential 250-base sequence fragments, incrementing by 25 bases (midpoints indicated in x axis). Changes in sequence orders resulting from changes in phylogeny at the 70% bootstrap level are shown. Sequences are color coded by genotype, as indicated by labels in left margins: genotype A, red; B, green; C, yellow; D, blue; E, purple; F, pink; G, pale yellow; H, brown; chimpanzee (Ch), light blue; woolly monkey (outgroup on line 1), black. The boundaries of sequence groupings with 70% or greater bootstrap support are indicated with black horizontal lines; for clarity, only clades between genotypes (except for group I) and with six or more members were demarcated. For comparison, the TreeOrder Scan was aligned with a scale genome diagram of HBV (upper panel).

A recombination analysis method based on stepwise phylogenetic tree construction using the programs SEQBOOT, DNADIST, NEIGHBOR, and CONSENSE in the Phylip package was first brought out by Simmonds, 2005 [18]. Two sequences (AY236161 and AF461043) were used to evaluate the effectiveness of the reference groups derived from all HBV sequences we collected from GenBank up to April 2009. Changes in genotype grouping, potentially arising from recombination, are characterized by abrupt changes in grouping scores of the sequence, as shown for AY236161 and AF461043. The curvilinear trend on the plots was almost the same as the plots reported [18] before. Because GroupScan calculation is time consuming, nearly all of the available HBV sequences have been submitted to GroupScan and the results were collected as a database for web service (available at http://nidvd.xmu.edu.cn/qgenotype). The service includes GenBank ID query and a BLAST query based on NCBI BLAST technology, and the recombination plots can be loaded immediately via the two query methods without online phylogenetic calculation. For W29 the sequence, the curve attributed to genotype C occupies the most regions from 1600 to 3000. However, the first half of the sequence matches none of the human or ape HBV genotypes, and lacking a group score greater than 0.5 indicating an outgroup position and exhibiting no phylogenetic clustering with any reference group. In addition, the plots of Vietnamese and Laotian strains' sequences show almost the same behavior, except for a subtle difference of genotype composition in the first half of the sequence (Fig. 3). When composing the reference sequences, a distinct sequence (EU833891) submitted from Canada (unpublished) was found to have the same behavior as the I *group*, and this strains was also selected in our later analysis. To figure out the evolution relationship of these Vietnam strains and genotype C strains, TreeOrderScan was also conducted using all the above sequences. The distribution of different genotype clades in the tree order map is not exactly the same as the published result [18] mainly due to the initial group order and different quantity of reference genotype sequences, but the relative position and boundary indication of different genotypes clade is similar (Fig. 4). On the tree order map, there is no evidence showing any direct evolutionary relationship between the I *group* and genotype C strains except the last 600 bp embedded in genotype C clade.

### Amino Acid Comparisons of Different Genotypes and Subtypes

**Figure 5:**
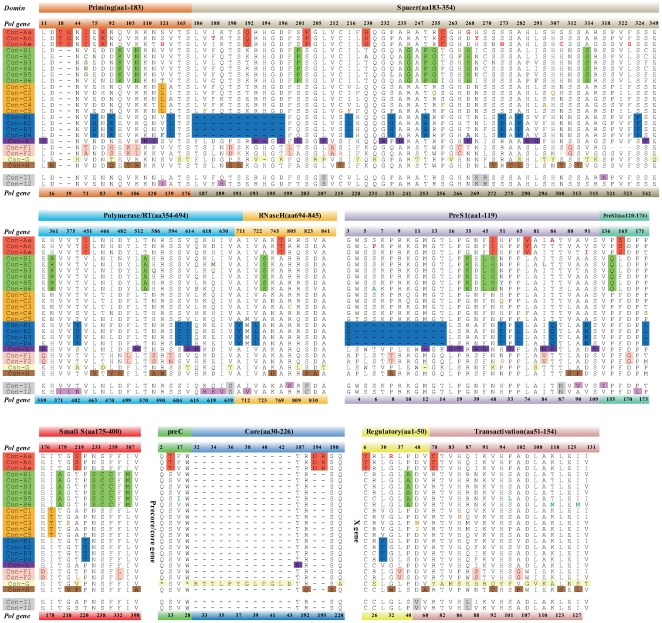
Consensus amino acid sequences of 4 gene products of HBV (*P*, *S*, *X* and *Pre-core/core*) were arrayed according to genotypes and subtypes, with identical columns excluded. Consensus sequences were derived from the 322 reference sequences. For page view limited, unique sites in subtype were also omitted and the sequence number of amino acids was indicated alternately on the top and bottom of the aligned matrix. Genotype-specific amino acids were highlighted with different colors and subtype-specific amino acids were only displayed in corresponding colored letters.

Fig. 5 lists the consensus amino acid sequence of all four HBV genome products of different genotypes and subtypes with identical columns excluded. Most of the 8 genotypes have several unique sites deduced from consensus sequences of various genotypes or subtypes. Genotype-specific amino acids scattered along all four gene products, and it seems that the space region of the P gene product deposits more special sites. These genotype-specific sites were also found in all five clones in our study. In the putative genotype I, 5 unique sites (S207, K269, R270, S639, F/C823) were found in the P gene, 2 unique sites (V48,L87) in the X gene, and 1 unique site (N87) in the preS gene. Genotype C shows only 2 distinct sites among all genome products whereas others have dozens.

### Phenotypic Characteristics of W29 Strain In Vitro and In Vivo

**Figure 6:**
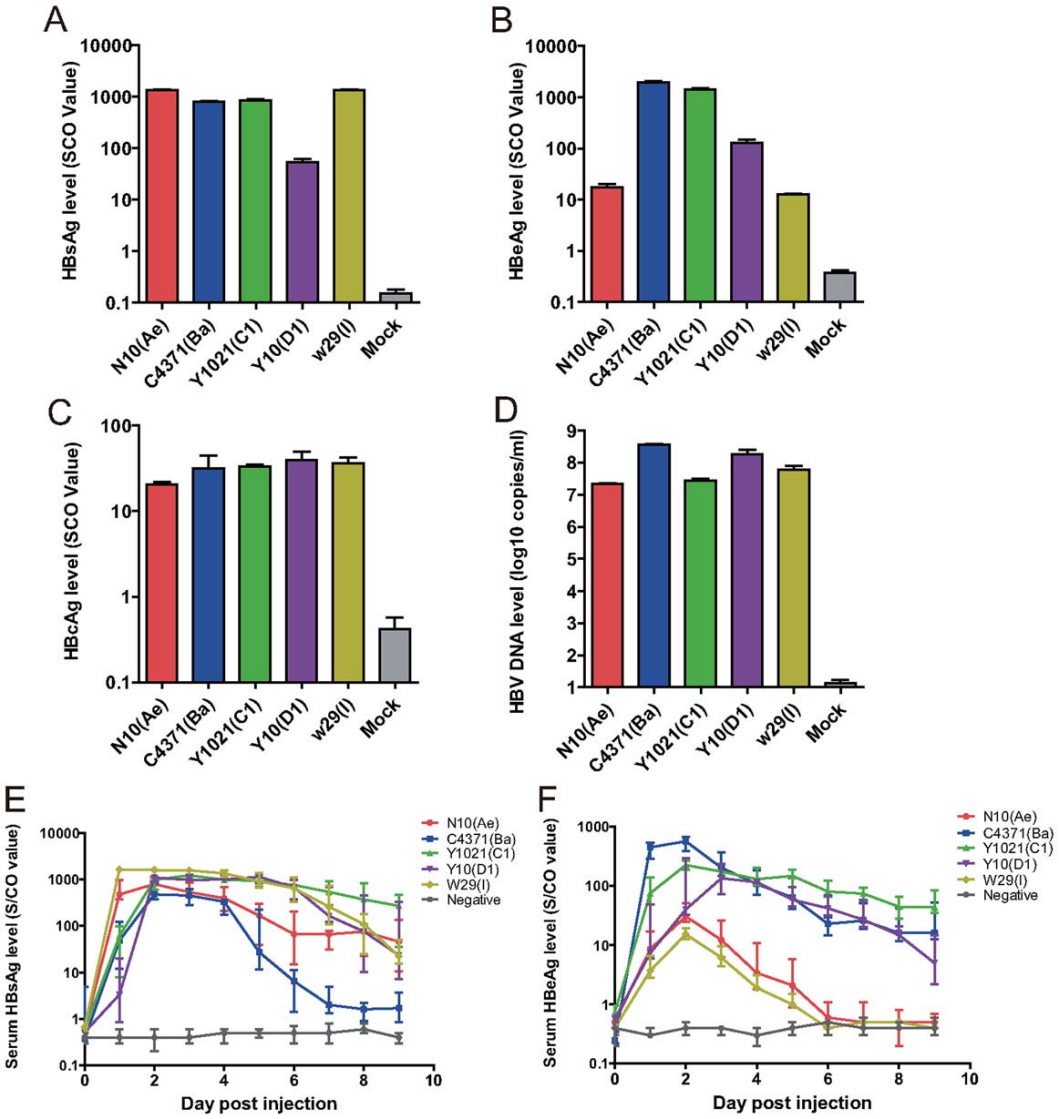
Comparison of expression levels of HBV markers between different genotypes *in vitro* and *in vivo*. Fig. a, b, c, d: HBsAg, HBeAg, HBV DNA and HBcAg levels in Huh7 cell culture supernatant. SEAP reporter plasmid was co-transfected with each HBV plasmid as an internal control. The SEAP activity of the first group (transfected with N10 (Ae) plasmid) was set as 1.0 and served as a reference, and the other groups were calculated as relative SEAP activity. The presented data are a ratio of actual value to the relative SEAP activity value. Fig. e, f: Dynamic curve of HBsAg and HBeAg expression levels in serums of hydrodynamic-injected mice. For each HBV plasmid, five mice were repeated. Due to the limited serum resources, each sample was diluted 10-fold for mensuration.

Five HBV 1.35-fold genome plasmids - N10 (Ae), C4371 (Ba), Y1021 (C1), Y10 (D1), W29 (I1) – all of which existed in different regions of China, were constructed for transfection and hydrodynamic injection. None of these genomes possessed mutations for G1896A in the precore region or A1762T/G1764A in the basic core promoter, which may interfere with the expression of HBeAg and the efficiency of pregenome encapsulation for replication. All five strains had detectable levels of HBsAg, HBeAg, HBcAg and HBV DNA in their transfected supernatants. The W29 (I1) and N10 (Ae) strains had a similar, significantly lower HBeAg expression level (P<0.01) than C4371 (Ba), Y1021 (C1) and Y10 (D1) in both of the Huh7 cellsand mice (Fig. 6). With regard to the HBsAg secretion, W29 (I1) had a similar level with N10 (Ae), C4371 (Ba) and Y1021 (C1), was and they were significant higher than Y10 (D1). In addition, all five species show similar peak HBsAg levels in mice serum. HBV DNA level and HBcAg levels in Huh7 cells exhibit no significant difference between the five strains. From an overview of the the results, the viral markers of W29 (I1) in Huh7 cells and mice had a similar level with N10 (Ae) but not Y1021 (C1), although it was more close to Y1021 (C1) in distance analysis.

## Discussion

This study includes almost all of the HBV sequences available from GenBank. The GroupScan results of two test sequences (AY236161 and AF461043) confirm that the selected reference sequences are representative and maximally informative phylogenetically. The plot for AB231908 is also the same as the previously published result [22], although we used a higher incrementing steps value. When composing the reference sequence dataset, nearly all of the HBV sequences available had been submitted to the GroupScan analysis and corresponding recombination plots generated. Besides one sequence reported from Canada, no other sequence was found to have the same behavior as the Vietnamese and Laotian sequences. Furthermore, these sequences fell into the same clade on both trees; one generated using the complete nucleotide sequence and the other using only P gene sequence. It can be clearly observed on these two trees that group I sequences respectively related to genotype A and C. This probably means that we'd better categorize group I sequences into an independent group distinct from any other known genotype than only as a subtype of genotype C. The tree order map also suggests that most regions of these sequences may not be attributed to the genotype C group. Using the limited SIMPLOT method, it had been proposed that the putative genotype I is a recombinant of A/C/G [20], but this conclusion was not supported by the newly developed method of GroupScan, which overcomes the limitation of SIMPLOT. In the recombination plot of W29, Vietnamese and Laotian strains, the group score of genotype A could be detected at a lower value (<0.5) in the region 1–1200 nt, but that is not sufficient evidence to conclude that this part of the genome may bury in the genotype A clade. In actuality, none of the known genotypes could be matched to this region of W29.

Generally, HBV genotype C includes several serotypes: *adrq+*, *adrq−*, *ayr*, *adw*, *adw2*, *ayw2*, *ayw3*, *adwr* [23]. We have checked all available group I sequences and found that all of them contain K or R on residue 122, K on residue 160 and P on residue 127. Thus the serotypes of group I may be narrowed down to *adw2* or *ayw2*. In genotype C strains, serotype *adw2* prevails in East Asia, Japan, and the Philippines and *ayw2* was only found in some Tibet strains in China [23]. It has been proposed by Muller that the group I sequences could be divided into two subgroups named I1 and I2 [17] and the divergence is also obvious in both phylogenetic trees generated in this study. Boundaries among group I in the tree order map (Fig. 4) were also retained to exhibit the divergence distribution along the complete genome. Interestingly enough, the I1 group falls into serotype *adw2* and the I2 group into serotype *ayw2*, without exception. As a result, the 122K/R could be employed as an identifier to distinguish the two subgroups.

It is well accepted that the expression level of HBV markers vary in different genotypes. In our cell culture results, secretion of HBsAg is uniform abundant in all genotype except genotype D, which agrees with the previous report [24]. But HBeAg expression is entirely different. W29 exhibits a lower secretion of HBeAg, at a level closest to genotype A, and much lower than that of genotype B/C or D. Corroborating results were also observed in the acute hydrodynamic injection mouse model, where HBsAg levels for all 5 genotypes were similar. It is the first report on HBsAg assay comparing genotype I with other genotype, the differences in HBsAg expression may be related to genotype specific differences in sensitivity. All five strains were submitted to GroupScan and the recombination plots generated were typical and representative of the known genotypes A to D (Data not shown). The 8 genotype-specific amino acids found on group I might contribute to their distinct characterization in *in vitro and in vivo* marker assay, causing 5 unique mutations located on polymerase related protein. Although the criteria of genotype differentiation do not include phenotype differences, the distinct phenotype of group I indicates an inevitable distance from genotype C.

It had been proposed for years that some strains from Vietnam and Laos might be grouped into a new genotype of HBV [7], [17]. However, the proposal is not yet widely accepted, because their sequences deviate less than 8% from genotype C [22]. The discovery of W29 expands the geographic distribution of this special group, as the original host lives in the northwest of China, and has never left the country. In addition, the EU833891 strains may also represent a new geography distribution. Phylogenetic analysis clearly show that W29 is most related to the putative genotype I. The average distance value between W29 and genotype C reach 8.4%, suggesting that W29 may represent a more distant clade from genotype C than the Vietnamese and Laotian strains do. When W29 was added to the presumed genotype I group, the average distance between genotype I and C is still as high as 8.0%, which just satisfies the well accepted criteria for definition of a new genotype. Using the GroupScan and TreeOrderScan methods developed by Simmond, we can conclude that the last half of the group I genome is only a partial analogue of genotype C's genome, and that the majority of the genome does not match any of the known HBV genotypes. In addition, the phenotypic characteristic of W29, which might also exist in Vietnamese and Laotian strains, account for the great disparity between genotype C and the putative genotype I. All things considered, this separate group of HBV strains might not be simply reduced to a regional or recombinant mutation, and the extending geographic distribution suggests that this group may have existed for a long time. Just before this article was submitted, another strain (FJ667206, partial genome) from France was reported as being clustered with the genotype I [25]. A suggested criterion raised by Schaefer also agreed to group these variants into a new genotype [26]. We believe that in the future, more strains will be discovered and add to this emerging new genotype.
